# A comprehensive review on the determination of enzymatic assay and nonenzymatic antioxidant activities

**DOI:** 10.1002/fsn3.1012

**Published:** 2019-04-02

**Authors:** Zainol Haida, Mansor Hakiman

**Affiliations:** ^1^ Department of Crop Science, Faculty of Agriculture Universiti Putra Malaysia Serdang Malaysia

**Keywords:** antioxidant, enzymatic assay, free radical, nonenzymatic assay

## Abstract

This review article presents a comprehensive review pertaining to antioxidants and various assays that determined enzymatic and nonenzymatic antioxidants. Antioxidants have gained attention at the global scale on its prominent beneficial roles that can fight against many chronic infirmities, including cancer and cardiovascular diseases. Many studies have investigated different types of samples, such as medicinal plants, fruits, and vegetables, by using various antioxidant assays. Antioxidants can be grouped into enzymatic and nonenzymatic antioxidants. To date, most studies had looked into nonenzymatic antioxidants due to lack of references on enzymatic antioxidant assays. Therefore, this review article depicts on seven assays of enzymatic antioxidants (superoxide dismutase, catalase, peroxidase, ascorbate peroxidase, ascorbate oxidase, guaiacol peroxidase, and glutathione reductase) and fifteen activities of nonenzymatic antioxidants (total polyphenol, total phenolic acids, total flavonoids, total ascorbic acid, anthocyanin content, DPPH scavenging activity, FRAP assay, hydrogen peroxide scavenging activity, nitric oxide scavenging activity, superoxide radical scavenging activity, hydroxyl radical scavenging activity, phosphomolybdate assay, reducing power, metal ion chelating activity, and β‐carotene), which are described in detail to ease further investigations on antioxidants in future.

## Abstract 2

The antioxidant can be classified into enzymatic and nonenzymatic. Human is being exposed to chronic diseases every day. Antioxidant is the best source to prevent or inhibit free radicals.

## 1 INTRODUCTION

It is a fact that human exposure to vast chronic ailments has never been higher than this present age. Free radicals are being generated at high quantities, particularly at metabolic and contact processes. Tissues and cells are damaged by oxidation as macromolecules (fatty acids, nucleic acids, and proteins) deteriorate (Gupta et al., 2014). Basically, the reaction that takes place between free radicals and electrons derived from other molecules harms the human body due to its negative effect on the enzyme system (Bjelakovic, Nikolova, Gluud, Simonetti, & Gluud, 2007). Hence, the complex systems of enzymatic and nonenzymatic antioxidants have the capability to address the deteriorating impacts of oxidants and free radicals that may lead to infirmity. An antioxidant is a type of compound that stabilizes, scavenges, and suppresses the generation of oxidants and free radicals. Therefore, consumption of antioxidants based on natural resources (greens, fruits, and herbs) may help to shield one from oxidants and free radicals without side effects.

All enzymatic and nonenzymatic antioxidant assays described in the review are based on UV–vis spectrophotometry method. Apart from spectrophotometry method for analysis of antioxidants, other methods such as electrochemical and chromatography methods have also been used. The electrochemical methods include cyclic voltammetry, superwave voltammetry, differential pulse voltammetry, coulometry, potentiometry, amperometry, and biamperometry. The chromatography methods include thin layer chromatography (TLC), liquid chromatography–mass spectrophotometry (LC‐MS), high‐performance liquid chromatography (HPLC), and gas chromatography. However, among all the methods mentioned above, the spectrophotometry method has been the most commonly used method because it is the easiest to handle and inexpensive to run when compared to the electrochemical and chromatography methods.

Spectrophotometry method can be performed by using a cuvette or a microplate reader. Analysis of antioxidant by using cuvette is usually conducted for small number of samples. The method is time consuming as only one sample is allowed to be read at one time which takes about 1–2 min. This method also requires high amount of reaction mixture due to large volume of cuvette (approximately 1.5–2 ml). In contrast, the use of a microplate is more convenient and time saving. It allows reading of 96 samples at one time. Presently, a microplate reader of spectrophotometer has been upgraded to allow reading of 384 samples at one time. By using a microplate reader, the volume of samples needed is significantly low (approximately 100–200 µl). Hence, the amount of reaction mixture needed to prepare is low, thus saving the use of chemicals.

As such, a number of methods have been proposed to quantify antioxidant content in many samples. With that, this paper presents a review pertaining to the common techniques employed to analyze both enzymatic and nonenzymatic antioxidants.

## 2 FREE RADICALS, REACTIVE OXYGEN SPECIES (ROS), AND REACTIVE NITROGEN SPECIES (RNS)

Free radicals are claimed to be harmful to humans because its unpaired electron(s) extracts electron(s) from other molecules in the body to gain stability, hence damaging DNA, proteins, and lipids. These free radicals, which can be found as nitrogen derived (RNS) or oxygen derived (ROS), have rather high reactivity and short life. Components that are present as free radicals in ROS are O_2_ (superoxide), HO^•^ (hydroxyl), HO_2_ (hydroperoxyl), ROO^•^ (peroxyl), and RO^•^ (alkoxyl), while those nonradicals refer to H_2_O_2 _(hydrogen peroxide), HClO (hypochlorous acid), O_3_ (ozone), and ^1^O_2_ (singlet oxygen). Meanwhile, NO^•^ (nitric oxide), NO_2_ (nitrogen dioxide), N_2_O_3_ (dinitrogen trioxide), and ONOO^−^ (peroxynitrite) are the free radicals derived from RNS (Ali et al., 2008; Evans & Halliwell, 1999).

The major source of ROS is the environment that is filled with car exhaust fume, cigarette smoke, ozone, and low‐wave electromagnetic and ultraviolet (UV) radiation. Meanwhile, some sources of the endogenous ROS are amino acids auto‐oxidation, respiratory burst by phagocytes, ischemia reperfusion injury, and mitochondrial electron transport chain. Hydroxyl radical (HO^•^), which is generated from H_2_O_2_ and O_2_ via Harber–Weiss reaction, refers to a damaging ROS with 10^−5^ s half‐life (Beauchamp & Fridovich, 1970). The H_2_O_2_ component, although with poor reactivity and relatively stable properties, can cross cell membranes easily and arrack various sites after conversion to HO. Besides, H_2_O_2_ produces free radicals with the existence of transition metal ions.

One essential RNS‐based free radical refers to nitric oxide (NO) as this component is one of the 10 smallest molecules that are naturally found in 30‐Da molecular weight. The processes of redox, substitution, addition, and chain termination reaction are closely associated to NO. According to Stamler (1994), the primary targets of NO are proteins that contain metals, intracellular thiol, and low molecular weight thiols (glutathione). The peroxynitrite (ONOO^−^) is also an essential antioxidant with a range of targets. The mechanism of ONOO^−^ is as follows: peroxidation of lipid, break of DNA strand, nitration of tyrosine, and, lastly, death of cell. Upon reaction with NO, ONOO^−^ produces NO_2_ and causes nitrosative stress, while generating N_2_O_2_ with more NO (Koppenol, Moreno, Pryor, Ischiropoulos, & Beckman, 1992).

Harmful oxidative stress, which can lead to chronic disease, is produced when RNS and ROS concentrations exceed the amount of antioxidants in the human body.

## 3 ANTIOXIDANTS

Oxidative stress can be addressed by antioxidants by delaying or preventing the oxidative chain reaction promulgation. Plant‐based antioxidants scavenge free radicals to inhibit tissue/cell damages, hence minimizing risk of chronic diseases (Soobrattee, Bahorun, Neergheen, Googoolye, & Aruoma, 2008). Besides, antioxidants donate their electrons in order to neutralize both exogenous and endogenous free radicals (Jacob, 1995).

Antioxidant components are composed of lipid‐soluble (hydrophobic) and water‐soluble (hydrophilic) substances. Plant‐based antioxidants are mostly hydrophilic, in which some instances are phenolics, flavonoids, superoxide dismutase (SOD), catalase (CAT), glutathione peroxidase (GPx), as well as uric, lipoic, benzoic, and ascorbic acids. On the other hand, hydrophobic antioxidants are linked to biological membrane functional, which are found in carotenoids, tocopherols, vitamin K, ubiquinone, and phospholipids.

Additionally, antioxidants are categorized depending on defense lines: preventive antioxidant as the initial line of defense, radical scavenging antioxidant in the second line of defense, and, lastly, repair and de‐novo enzymes as the third line of defense, as further elaborated:

- The first line of defense hinders the production of free radicals that leads to oxidative stress via enzymes, such as SOD, CAT, GPx, glutathione reductase, and several types of minerals, for example, selenium (Se), manganese (Mn), copper (Cu), and iron (Fe).
- The second line of defense inhibits the production of damaged species, apart from making the free radicals less harmful that further reduces damages caused by oxidative reaction. Some excellent scavengers of free radicals are vitamins E and C, flavonoids, and uric acid.
- The third line of defense serves to repair damaged DNAs, proteins, peroxides, and oxidized lipids, besides inhibiting the propagation of chain reaction in peroxyl lipid radical.

The classification of antioxidants depends on their catalytic action type, either enzymatic or nonenzymatic antioxidants. The enzymatic antioxidants possess certain cofactors and tend to be highly specific for substrate reactive species. Some instances of enzymatic antioxidants refer to SOD, CAT, and GPx. Meanwhile, those nonenzymatic antioxidants differ from the former as they do not possess any specific substrate and, hence, could nullify the negative effects of both RNS and ROS.

## 4 ENZYMATIC ANTIOXIDANT ASSAY

### 4.1 Superoxide dismutase assay

The enzyme extract was prepared by homogenizing fresh samples (200 mg) in 5 ml of 100 mM potassium phosphate buffer (pH 7.8) that contained 0.1% (v/v) Triton X‐100, 2% (w/v) polyvinyl pyrrolidone (PVP), and 0.1 mM EDTA. Next, the extract was filtered and centrifuged at 22,000 × *g* for 10 min at temperature 4°C. The resultant supernatant was collected and dialyzed by using cellophane membrane tubing for 240 min against cold extraction buffer. The remaining extract was used for enzyme assay.

The final volume of incubation mixture is 3 ml that contains 50 mM of potassium phosphate buffer (pH 7.8), 45 µM of methionine, 20 µM of potassium cyanide, 84 µM of nitroblue tetrazolium (NBT), and 5.3 mM of riboflavin. The amount of homogenate added to the mixture is kept under one unit of enzyme so as to ensure high accuracy. The mixture is incubated at 25°C with the presence of 15 W fluorescent lamps in an aluminum foil‐lined box. After 10 min of exposure to light, the reduction in NBT is measured at absorbance of 600 nm. Absence of enzyme is indicated by the highest reduction. One unit of enzyme activity is defined as the amount of enzyme that leads to 50% inhibition of NBT reduction (Misra & Fridovich, 1972).

### 4.2 Catalase assay

Extraction of catalase assay was prepared by homogenizing fresh samples (200 mg) in 5 ml of 50 mM Tris‐NaOH at pH 8.0 that contained 0.5% (v/v) Triton X‐100, 2% (w/v) PVP, and 0.5 mM EDTA. The homogenate was centrifuged for 10 min at 4°C at 22,000 × *g*, and the resultant supernatant was dialyzed prior to enzyme assay.

Catalase assay can be conducted by adhering to the method suggested by Aebi (1984). One milliliter of reaction mixture containing 50 mM of potassium phosphate buffer (pH 7.0) and 250 µl of enzyme extract is initiated by adding 60 mM of hydrogen peroxide. The absorbance is measured by using a spectrophotometer at an absorbance rate of 240 nm for 3 min. The H_2_O_2_ decomposition is calculated by using extinction coefficient of 39.4 mM^−1^ cm^−1^. One unit of activity is equivalent to 1 mM of H_2_O_2_ degraded per minute and is expressed as unit per milligram of protein.

### 4.3 Peroxidase assay

Two grams of samples had been homogenized with 10 ml of 0.1 M phosphate buffer (pH 6.0). The extract was filtered through cheesecloth and centrifuged for 30 min at 12,000 × *g*. The resultant supernatant was collected and heated at 65°C for 3 min to inactivate any catalase present in the extract.

Peroxidase assay is prepared by performing the guaiacol oxidation method as depicted by Britton and Mehley (1955). The final reaction mixture (3 ml) in the test tube consists of 10 mM of potassium phosphate buffer (pH 7.0), 8 mM of guaiacol, and 100 µl of enzyme extract, in which 2.75 mM of hydrogen peroxide is added to initiate the reaction. Increment in absorbance measured at 470 nm within 30 min indicates the formation of tetraguaiacol. The change in absorbance per min and specific activity as enzyme units per mg soluble protein with extinction coefficient 6.39 mM^−1^ cm^−1^ refers to a unit of peroxidase activity. The enzyme activity is expressed as unit per milligram of protein.

### 4.4 Ascorbate peroxidase assay

The enzyme assay was extracted from 200 mg of samples. Next, the samples were homogenized in 50 mM potassium phosphate buffer (5 ml, pH 7.8) that contained 1 mM ascorbic acid, 1 mM phenylmethane sulfonyl fluoride (PMSF), and 1% PVP. The reaction mixture was centrifuged at 22,000 × *g* at 4°C for 10 min. The resultant supernatant was collected and dialyzed prior to enzyme assay.

The reaction mixture that contains 100 mM of Tris‐acetate buffer (pH 7.0), 2 mM of ascorbic acid, and enzyme extract is added with 2 mM of hydrogen peroxide to initiate the reaction. The decrease in absorbance rate is measured by using a spectrophotometer at 290 nm for 100 s. The extinction coefficient 2.8 mM^−1^ cm^−1^ is used to calculate the reaction. The specific enzyme activity is expressed as unit per milligram of protein (Ali, Hahn, & Paek, 2005).

### 4.5 Ascorbate oxidase assay

The plant sample tissue was extracted in 20 mM potassium phosphate (pH 7.4), 1.5% PVPP, and 0.5 mM PMSF. After that, the mixture was homogenized with Polytron, incubated on ice for 20 min, and vortexed for every 2‐min interval. Next, the mixture was centrifuged at 15,000 × *g* at 4°C for 15 min. The resultant supernatant was collected and dialyzed prior to enzyme assay.

The final reaction mixture consists of 1.0 ml that is comprised of 20 mM of potassium phosphate buffer (pH 7.0) and 2.5 of mM ascorbic acid. The 10 µl of enzyme extract is added to initiate the reaction. Due to ascorbate oxidation, the decrease in absorbance is monitored for 3 min at an absorbance rate of 265 nm and calculated by using extinction coefficient, 14 mM^−1^ cm^−1^ (Diallinas et al., 1997).

### 4.6 Guaiacol peroxidase assay

The enzyme extract for determination of guaiacol peroxidase assay was performed by homogenizing 200 mg of fresh samples in 5 ml of cold 50 mM sodium phosphate buffer at pH 7.0. Next, the dialyzed enzyme extract was used for assay after being centrifuged at 22,000 × *g* for 10 min.

The assay mixture (5 ml) contained 2 mM H_2_O_2_, 9 mM guaiacol, 40 mM sodium phosphate (pH 6.1), and 50 µl enzyme. The increment in absorbance was measured at 420 nm and calculated by using extinction coefficient of 26.6 mM^−1^ cm^−1^ for 2 min with 30‐s interval. The outcomes are expressed as unit per milligram of protein (Egley, Paul, Vaughn, & Duke, 1983).

### 4.7 Glutathione reductase assay

Glutathione reductase assay was carried out by adhering to the method depicted by Schaedle and Bassham (1977). The enzyme extract was prepared prior to enzyme assay. Briefly, 200 mg of fresh samples was homogenized by using chilled mortar and pestle in 5 ml of 50 mM Tris‐HCl buffer at pH 7.6. The resultant supernatant was collected after being centrifuged at 22,000 × *g* for 4 min and dialyzed prior to enzyme assay.

The final reaction mixture (1 ml) was composed of 200 µl enzyme extract, 50 mM Tris‐HCl buffer (pH 7.6), 1 mM glutathione disulfide (GSSG), 0.15 mM NADPH, and 3 mM MgCl_2_. A decrease in NADPH absorbance was observed at 340 nm. The specific activity of enzyme is expressed as unit per milligram of protein.

## 5 NONENZYMATIC ANTIOXIDANTS ASSAY

### 5.1 Total polyphenol content

Polyphenols are polyhydroxylated phytochemical that are synthesized by plants and have many benefits to the human health. Polyphenols have abilities to trap and to scavenge free radicals by donating hydrogen ion to stabilize the free radicals. In addition, polyphenols can regulate nitric oxide, induce apoptosis, inhibit cell proliferation and angiogenesis, and prevent high blood pressure, apart from possessing anti‐aging, anti‐bacterial, and anti‐tumor properties. The two major subclasses of polyphenols are phenolic acids and flavonoids (Arts & Hollman, 2005; Yazaki, Sasaki, & Tsurumaru, 2009).

The total polyphenols content in the plant can be determined by employing the method suggested by Marinova, Ribarova, and Atanassova (2005). A total of 10 µl of sample extract are mixed with 2.5 ml of 10‐fold of diluted Folin–Ciocalteu reagent. After 5 min, 2.5 ml of 7% sodium carbonate is added and the mixture is incubated at room temperature. After an hour of incubation, the absorbance of reaction mixture is measured at 725 nm. The total polyphenol content is expressed as milligram gallic acid equivalents per gram of samples.

### 5.2 Total phenolic acids

Phenolic acids are hydroxylated that derive from benzoic and cinnamic acids. Hydrobenzoic acid is mainly present in the form of glucosides in foods, while hydrocinnamic acid, such as *p*‐coumaric, caffeic acid, and ferrulic acid, is mostly found in food as simple esters (Mattila & Kumpulainen, 2002).

Total phenolic acids can be determined by adhering to the method proposed by Singleton and Rossi (1965), which is known as the Folin–Ciocalteu phenol reagent technique. Briefly, 1 ml of sample extract is added into a test tube that contains 9 ml of distilled water. Then, 1 ml of Folin–Ciocalteu phenol reagent is added to it and the mixture is mixed thoroughly via vortex. After 5 min, 10 ml of 7% sodium carbonate is added. Next, 4 ml of distilled water is added and the mixture is adjusted to 25 ml of final volume. The reaction mixture is incubated for 90 min at room temperature, and the absorbance is measured at 750 nm. The total phenolic acids are expressed as milligram of gallic acid equivalents per gram of samples.

### 5.3 Total flavonoids

Flavonoids are widely found in plants, and they consist of a large group of polyphenolic compounds that can be characterized by benzo‐y‐pyrone structure. Flavonoids are low molecular phenolics, and they can be divided into several subclasses, such as flavones, flavanones, isoflavones, anthocyanins, flavanols, and flavonols. The most crucial function of flavonoids is their antioxidant activity. Flavonoids can scavenge various oxidizing species, including superoxide anion, hydroxyl or peroxyl radicals by quenching the singlet oxygen. Flavonoids can also act as an inhibitor in oxidation of low density lipoprotein (LDL), anti‐bacterial, and anti‐fungal, besides preventing malaria (Harborne & Williams, 2000; Subramanian, Stacey, & Yu, 2007).

The content of total flavonoids can be determined based on the method depicted by Marinova et al. (2005) by employing the aluminum chloride colorimetric technique. Briefly, 1 ml of sample extract is added into a test tube containing 4 ml of distilled water. After that, 0.3 ml of 5% sodium nitrite is added. After 5 min, 0.3 ml of 10% aluminum chloride is added into the mixture. At the sixth min, 2 ml of 1 M sodium hydroxide is added. Next, the mixture is adjusted to 10 ml by adding 2.4 ml of distilled water and mixed thoroughly with a vortex machine. The absorbance of the reaction mixture is measured at 510 nm. The total flavonoid content of the extract is expressed as milligram rutin equivalents per gram of samples.

### 5.4 Total ascorbic acid

Ascorbic acid or vitamin C is an organic acid that is the most crucial vitamin for human nutrition. Ascorbic acid is synthesized in mitochondria and transported to other cell through proton–electron chemical gradient or through facilitated diffusion. It also has the ability to scavenge many types of free radicals. The main sources of ascorbic acid are fruits and vegetables. Ascorbic acid can fight against chronic diseases, such as cardiovascular diseases and certain types of cancer (Aro & Ohad, 2003; Borland et al., 2006; Gil, Tomás‐Barberán, Hess‐Pierce, & Kader, 2002).

The total ascorbic acid can be determined by using 1% phosphate citrate buffer (Davies & Masten, 1991). Briefly, fresh plant sample is extracted by using chilled pestle and mortar with the addition of 1% phosphate citrate buffer (pH 3.5). Next, the sample is homogenated and centrifuged at 14,000 x *g* for 10 min at 4°C. The supernatant is collected, and 1.72 mM of 2,6‐dichloroindophenol (2,6‐DCPIP) is added. The absorbance is immediately measured after mixing at 518 nm by using a spectrophotometer.

### 5.5 Anthocyanin content

Anthocyanins are chemicals that have a single aromatic structure known as cyaniding, which refers to a widely distributed pigment group found in the plant kingdom. The colors range from red to blue of the visible spectrum due to the water‐soluble pigments. The colors of these substances are varied due to addition or removal of hydroxyl groups or because of methylation or glycosylation (Harborne & Williams, 2000; Sakakibara, Honda, Nakagawa, Ashida, & Kanazawa, 2003).

Determination of anthocyanin content can be carried out based on the technique proposed by Bharti and Khurana (2003). Briefly, fresh leaves are added into 10 ml of acidic methanol (1% v/v HCl) and the mixture is incubated overnight. To partition anthocyanin from chlorophyll, 10 ml of chloroform and 9 ml of double deionized water are added. The test tube is shaken gently, and the mixture is allowed to settle. The absorbance of the reaction mixture is measured at 505 nm.

### 5.6 DPPH scavenging activity

Molecule 1,1‐diphenyl‐2‐picrylhydrazyl (DPPH) is classified as a stable free radical by virtue of the delocalization of the spare electron over the molecule as a whole that prevents it from dimerize. When a solution of DPPH is mixed, it donates hydrogen atom that reduces its form and loses its violet color.

The DPPH radical scavenging activity can be carried out by preparing 1 ml of sample extract in a test tube. Next, 2 ml of 1 mM of methanolic DPPH is added. The solution is mixed thoroughly and incubated for 30 min at 37°C. The blank sample is prepared without adding any standard or sample extract. The change in absorbance is measured at 515 nm, and the percentage of inhibition is calculated (Alhakmani, Kumar, & Khan, 2013).

$$\text{Inhibition}\left(\%\right)=\left[\left({\text{A}}_{515}\;\text{Control}-{\text{A}}_{515}\;\text{Sample}\right)/{\text{A}}_{515}\;\text{Control}\right]\times 100$$

### 5.7 Ferric reducing‐antioxidant power (FRAP) assay

The FRAP assay measures the reduction of ferric iron and 2,3,5‐triphenyl‐1,3,4‐triaza‐azoniacyclopenta‐1,4‐diene chloride to blue ferrous complex by antioxidants under acidic condition (pH 3.6). The FRAP unit is the reduction of one mole of Fe (lll) to Fe (ll).

The FRAP assay can be prepared based on the method described by Wong, Leong, and Koh (2006). Briefly, 200 µl of sample extract is added with 3 ml of FRAP reagent that is prepared with a mixture of 300 mM of sodium acetate buffer (pH 3.6), 10 mM of 2,4,6‐tri(2pyridyl)‐s‐triazine (TPTZ) solution, and 20 mM of FeCl.6H_2_O at the ratio 10:1:1. The reaction mixture is incubated for 30 min at 37°C. Increment in absorbance is measured at 593 nm, and the percentage of inhibition (antioxidant) is calculated.

$$\text{Inhibition}\left(\text{antioxidant}\right)\left(\%\right)=\left[\left({\text{A}}_{593}\;\text{Sample}-{\text{A}}_{593}\;\text{Control}\right)/{\text{A}}_{593}\;\text{Sample}\right]\;\times 100$$

### 5.8 Hydrogen peroxide (H_2_O_2_) scavenging activity

Hydrogen peroxide is found naturally at low concentration levels in air, water, human body, plants, foods, and microorganism. H_2_O_2 _may enter the human body by inhalation of vapor or mist and through eye or skin contact. Hydroxyl radicals (OH^•^) are the by‐products of H_2_O_2 _decomposed into oxygen and water that initiate lipid peroxidation and damages to DNA.

In order to determine the scavenging activity of hydrogen peroxide, 40 mM of hydrogen peroxide solution is prepared in 50 mM phosphate buffer (pH 7.4) and the absorbance is measured at 230 nm. Next, 1 ml of sample extract or standard is added with 2 ml of hydrogen peroxide solution. After 10 min, the absorbance is measured against blank solution. The blank solution is prepared with phosphate buffer without adding hydrogen peroxide. Then, the percentage of hydrogen peroxide scavenge can be calculated (Nabavi, Ebrahimzadeh, Nabavi, Hamidinia, & Bekhradnia, 2008).

$${\text{H}}_{2}{\text{O}}_{2}\;\text{scavenge}\left(\%\right)=\left[\left({\text{A}}_{230}\;\text{Control}-{\text{A}}_{230}\;\text{Sample}\right)/{\text{A}}_{230}\;\text{Control}\right]\times 100$$

### 5.9 Nitric oxide scavenging activity

NO^•^ is generated in biological tissues by specific NO synthase that metabolizes arginine to citrulline via five‐electron oxidative reaction. At a physiological pH of 7.2, the sodium nitroprusside compound is decomposed into aqueous solution and generates NO^•^. Stable products (nitrate and nitrite) are produced when NO^• ^reacts with oxygen under aerobic condition, which can be determined by using the Griess reagent.

The scavenging activity of nitric oxide can be determined based on the method proposed by Marcocci, Maguire, Droylefaix, and Packer (1994). Briefly, 2 ml of 10 mM of sodium nitroprusside is prepared in 0.5 ml of phosphate buffer saline (pH 7.4). Next, 0.5 ml of sample extract is added and incubated at 25°C. After 150 min of incubation, 0.5 ml of Griess reagent (1% sulfanilamide, 2% H_3_PO_4_, and 0.1% naphthylethylenediamine dihydrochloride) is added to 0.5 ml of incubated solution. The reaction mixture is re‐incubated for 30 min at room temperature. The rate of absorbance is measured at 546 nm, and the inhibition percentage is calculated.

$$\text{NO inhibition}=\left[\left({\text{A}}_{546}\;\text{Control}-{\text{A}}_{546}\;\text{Sample}\right)/{\text{A}}_{546}\;\text{Control}\right]\times 100$$

### 5.10 Superoxide radical scavenging activity

Although the superoxide anion is a weak oxidant, it can ultimately produce powerful and dangerous hydroxyl radicals and singlet oxygen, which contributes to oxidative stress.

This assay can be prepared based on the method developed by Robak and Gryglewski (1988). The final mixture of 16 mM Tris‐HCl buffer at pH 8.0 (3 ml) is comprised of 0.3 mM of NBT (0.5 ml), 0.936 mM of NADH solution (0.5 ml), and 1 ml of sample extract. The mixture reaction is initiated by adding 0.5 ml of 0.12 mM phenazine methosulfate (PMS), and the mixture is incubated at 25°C for 5 min. The absorbance is measured at 560 nm against blank sample, and the percentage of inhibition is calculated.

$$\text{Inhibition}\left(\%\right)=\left(1-{\text{A}}_{560}\;\text{Sample}/{\text{A}}_{560}\;\text{Control}\right)\times 100$$

### 5.11 Hydroxyl radical scavenging activity

The hydroxyl refers to a dangerous free radical that belongs to ROS. Hydroxyl radical reacts with polyunsaturated fatty acid moieties of cell membrane phospholipids, which can inflict damages to the human cell.

The ability displayed by hydroxyl radicals to scavenge can be determined by adhering to the method described by Kunchandy and Rao (1990). The final mixture (1.0 ml) contains 28 mM of 2‐deoxy‐Dribose (100 µl) in 20 mM of potassium phosphate buffer (pH 7.4), 500 µl of sample extract, 1.04 mM of EDTA (200 µl) and 200 µM of FeCl_3_ (1:1 v/v), 1.0 mM of H_2_O_2_ (100 µl), and 1.0 mM of ascorbic acid (100 µl). Next, the mixture is incubated for an hour at 37°C. After that, 1.0 ml of 2.8% trichloroacetic acid and 1.0 ml of 1% thiobarbituric acid are added and the mixture is re‐incubated for 10 min at 100°C. The solution mixture is cooled on ice, and the absorbance rate is measured at 532 nm. After that, the extract is substituted with distilled water as blank, while the sample blank contains the sample solution, but without deoxyribose. Lastly, the inhibition percentage is calculated.

$$\text{Inhibition}\left(\%\right)=\left[{\text{A}}_{532}\;\text{Blank}-\left({\text{A}}_{532}\;\text{Extract}-{\text{A}}_{532}\;\text{Sample blank}\right)/{\text{A}}_{532}\;\text{Blank}\right]\times 100$$

### 5.12 Phosphomolybdate assay (total antioxidant capacity)

The total antioxidant capacity assay can be used to determine the capacity of antioxidants through the formation of phosphomolybdenum complex. The assay is based on the reduction of Mo (Vl) to Mo (V) by using sample analyte, which subsequently produces a green phosphate Mo (V) complex at acidic condition.

Briefly, 0.1 ml of the sample extract is added to 0.3 ml of reagent solution that contains 0.6 M of sulfuric acid, 28 mM of sodium phosphate, and 4 mM of ammonium molybdate. The test tube is covered and incubated at 95°C for 90 min. After that, the mixture is cooled at room temperature and the absorbance is measured at 695 nm. The blank solution that functions as control contains both the reagent solution and the solvent. The total antioxidant capacity is calculated (Priya, Rajaram, & Suresh‐kumar, 2012).

$$\text{Total antioxidant capacity}\left(\%\right)=\left[\left({\text{A}}_{695}\;\text{Control}-{\text{A}}_{695}\;\text{Sample}\right)/{\text{A}}_{695}\;\text{Control}\right]\times 100$$

### 5.13 Reducing power

The principle of this method is based on the increment of absorbance that indicates an increase in the antioxidant activity. Compounds with reducing power ability are electron donors and, hence, possess the ability to reduce the oxidized intermediate process of lipid peroxidation.

Briefly, the reaction mixture contains 2.5 ml of sample extract, 2.5 ml of 0.2 M sodium phosphate buffer (pH 6.6), and 2.5 ml of 1% potassium ferric cyanide. Next, the reaction mixture is incubated at 50°C. After 20 min of incubation, 2.5 ml of 10% (w/v) trichloroacetic acid and the mixture is centrifuged at 1,000 x *g *for 10 min. Then, 2.5 ml of the upper layer in the supernatant is collected and mixed with 2.5 ml of distilled water and 0.5 ml of 0.1% ferric chloride. The absorbance of reaction mixture is measured at 700 nm against blank, which consists of all reagents without the sample extract (Gulcin, Tel, & Kirecci, 2008).

### 5.14 Metal ion chelating activity

Formation of chelates with Fe^2+^_, _ferrozine can generate a complex that is red in color. Nevertheless, the fading of red shade in ferrozine‐Fe^2+^ complexes is only restricted in the presence of other chelating agents. The measurement of the reduced color determines the chelating activity that competes with ferrozine for ferrous ion.

Briefly, 800 µl of sample extract is added to 2 mM of ferrous chloride (100 µl). In order to initiate the reaction, 5 mM of ferrozine (400 µl) is added and the mixture is incubated for 10 min at room temperature. The absorbance of the mixture is measured at 562 nm against the blank sample. Citric acid or EDTA can serve as control, whereas distilled water can function as blank. The capacity to chelate ferrous ion is calculated (Prieto, Pineda, & Aguilar, 1999).

$$\text{Chelation}\left(\%\right)=\left[1-\left({\text{A}}_{562}\;\text{Sample}/{\text{A}}_{562}\;\text{Control}\right)\right]\times 100$$

### 5.15 β‐carotene/linoleic acid bleaching

β‐carotene bleaching is one of the fastest methods that can be employed to screen antioxidants based on the principle that linoleic acid, which is an unsaturated fatty acid, gets oxidized by ROS that is produced by oxygenated water. The produced resultant can initiate the oxidation of β‐carotene, thus leading to discoloration.

In brief, β‐carotene (0.2 mg/ml) is dissolved in chloroform and added into a round bottom flask, along with 20 µl of linoleic acid and 200 µl of Tween 20. A volume of 200 µl of the sample extract, either control or standard, is added. The mixture is evaporated at room temperature to dry under vacuum. Next, 50 ml of distilled water is added and the mixture is shaken to form a liposome solution. The initial absorbance (Abs^0^) of the mixture is measured at 470 nm. After that, the remaining solution is placed in water bath at 50°C for 120 min and the absorbance is recorded at 470 nm (Abs^120^). As for the control solution, 80% of methanol excluding sample extract can be used. The percentage of antioxidant activity is calculated (Dastmalchi, Dorman, Laakso, & Hiltunen, 2007).

$$\text{Antioxidant}\left(\%\right)=\left[1-\left({\text{Abs}}^{0}\;\text{Sample}-{\text{Abs}}^{120}\;\text{Sample}\right)/\left({\text{Abs}}^{0}\;\text{Control}-{\text{Abs}}^{120}\;\text{Sample}\right)\right]\times 100$$

## 6 PRECAUTIONS, REPEATABILITY, AND LIMIT OF DETECTION

Performing an in vitro antioxidant assay requires several precautions in order to reduce error. Both the enzymatic and nonenzymatic in vitro antioxidant assays need to be conducted in a dark room. An antioxidant is a sensitive compound that easily deteriorates when exposed to light. Alternative to using a dark room, preparation of samples also can be performed by covering the flasks with aluminum foil or by using amber glass bottles. Enzymatic antioxidant assay needs to be performed in a cold room (approximately 4°C) due to higher rate of fluctuations. In an in vitro antioxidant assay, the reagents used need to be freshly prepared as the reactivity of the reagents decreases with time. Preferably, extraction of samples needs to be prepared fresh prior to carrying out the assay. Although samples can be stored in a freezer (at −4 to −80°C) for several days, antioxidant activity is generally lower as compared to freshly prepared samples.

Repeat of in vitro antioxidant assay is usually conducted to increase precision and yield more accurate results. In order to establish repeatability, an in vitro antioxidant assay needs to be conducted in the same place, using the same procedures and instruments. Environmental conditions such as room temperature and light intensity need to be adjusted to same. An in vitro antioxidant assay for each sample needs to be repeated for at least three times, and an average reading is calculated. With the average of readings, a standard deviation and a standard error can be obtained. Significance of results is determined by conducting statistical analysis. Comparison between samples of antioxidant activity can be analyzed by calculating IC_50_ values. IC_50_ is defined as the concentration of sample required to exhibit 50% of free radical inhibition. The lower the amount of sample required to inhibit 50% of free radicals, the greater the antioxidant activity of the sample.

Limit of detection (LOD) is defined as the lowest amount or concentration of test samples that can be detected from zero. Prior to calculating the LOD, a calibration curve of sample needs to be plotted and linear equation be obtained. The limit of detection is calculated by using the formula (Yilmaz, Sadikoglu, Saglikoglu, Yagmur, & Askin, 2008):

Limit of detection=3s/m

where *s* is the standard deviation and *m *is the slope.

## 7 CONCLUSION

At present, researches pertaining to antioxidant compounds from plant sources have attracted attention across the globe due to its therapeutically and pharmacologically potent properties with low or no side effects to human. Besides, the increasing uses of chemicals in food productions, pollution, smoking, and synthetic medicine appear to increase the chances of free radicals based diseases. Plants are the biggest source of antioxidants, and they are sufficient to be used as medicine to fight against harmful diseases. This review article looked into enzymatic and nonenzymatic assays to evaluate antioxidants. Hence, this review article serves as a comprehensive reference for those keen in studies pertaining to antioxidants.

## CONFLICT OF INTEREST

The authors declare no conflict of interest.

## ETHICAL STATEMENT

This study does not involve any human and animal testing.
